# Protocol for analyzing emergence dynamics of diabetes with obesity using numerical continuation and bifurcation analysis

**DOI:** 10.1016/j.xpro.2024.102880

**Published:** 2024-02-12

**Authors:** Vehpi Yildirim, Peter M.A. Sloot

**Affiliations:** 1 Department of Public and Occupational Health, Amsterdam University Medical Centers, University of Amsterdam, 1081 BT Amsterdam, the Netherlands; 2 Institute for Advanced Study, University of Amsterdam, 1012 GC Amsterdam, the Netherlands; 3 Computational Science Lab, University of Amsterdam, 1098 XH Amsterdam, the Netherlands

**Keywords:** Biophysics, Metabolism, Systems biology, Computer sciences

## Abstract

Type 2 diabetes (T2D) is a multifactorial disease that slowly and inconspicuously progresses over years. Here, we present a protocol for analyzing slow progression dynamics of T2D with obesity. We describe steps for using software to exploit the differences between the timescales of the metabolic variables and using numerical continuation and bifurcation analysis. We detail procedures to analyze bi-stable system dynamics and identify the thresholds that separate healthy and diabetic states.

For complete details on the use and execution of this protocol, please refer to Yildirim et al. (2023).^1^

## Highlights

• Protocol analyzes emergence dynamics of diabetes with obesity
• Steps described to perform numerical continuation and bifurcation analysis
• Guidance on using software packages to translate XPPAUT models into Python

## Abstract (editor highlights)

Publisher’s note: Undertaking any experimental protocol requires adherence to local institutional guidelines for laboratory safety and ethics.

## Graphical abstract

**Figure:**
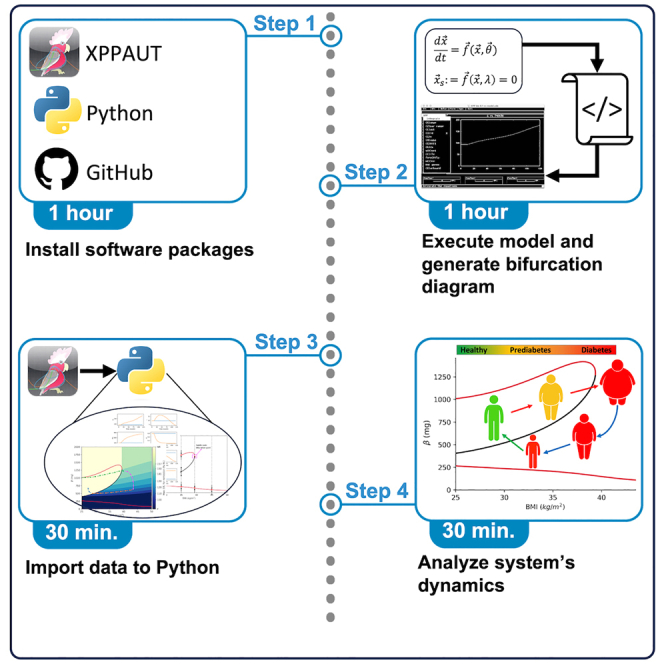

## Before you begin

This section introduces necessary software packages and instructions for their installation. Here, we use the protocol to analyze the slow progression dynamics of T2D with obesity using a recently published model.^1^ However, the protocol can easily be adapted for analyzing behaviors of other dynamical systems, whose dynamics are described with ordinary differential equations (ODEs). Although, some software packages are developed specifically for this protocol, they can easily be adapted for analyzing other dynamical systems.

### Download and install XPPAUT

**Timing: 20 min**

XPPAUT is a software package for simulating and analyzing mathematical models of dynamical systems.^2^ XPP is another commonly used name for XPPAUT, which includes an implementation of AUTO numerical continuation software.^3^ Together with AUTO, XPPAUT provides a very robust and powerful platform for simulating and analyzing nonlinear dynamical systems.

1. Go to https://sites.pitt.edu/∼phase/bard/bardware/xpp/xpp.html to download the latest version of XPPAUT, which is freely available under GNU public license.
  a. Choose the suitable version for your operating system and download the folder that contains XPPAUT software. XPPAUT is available for Windows, Linux and MacOS operating systems.
  b. Follow the operating system specific step-by-step installation instruction.

**CRITICAL:** For Apple users, different packages exist for Intel ad Apple silicon machines. Make sure that you choose the correct version.

2. Go to https://sites.pitt.edu/∼phase/bard/bardware/xpp/xpp.html, and install an X-window graphical server that is suitable for your operating system following online instructions.

***Note:*** The current version of XPPAUT runs on the X-window graphical environment. Installation guidelines for X-window servers for different operating systems are available online at the link given above.

### Download and install anaconda python platform

**Timing: 20 min**

One of the easiest ways to install Python and manage Python packages is through Anaconda platform, which provides a variety of software collections for scientific computing and machine learning applications. Anaconda also provides an advanced package and virtual environment control system (conda).

3. Go to https://www.anaconda.com/.
  a. Download the Anaconda version that is suitable for your operating system.
  b. Install Anaconda following operating system specific instruction.
    ***Note:*** Anaconda installation comes with Python along with a variety of packages such as NumPy, SciPy, Pandas and Matplotlib, which are necessary for this protocol. Anaconda platform can be used through both command prompt and an interactive desktop application (anaconda-navigator).
  c. Locate the executable app shortcut located in your applications (programs) list, and start anaconda-navigator.

### Download the model code and computer programs

**Timing: 5 min**

The protocol is implemented for a recently published computational model that describes the dynamics of the obesity driven progression of type 2 diabetes and remission through weight loss.^1^ The model describes system dynamics by a system of nonlinear ordinary differential equations (ODEs). An XPPAUT implementation of the model and other necessary programs are freely available under MIT license on GitHub repository.

4. Go to https://github.com/vehpi/T2D_str_pr.git and download the folder that contains the computer codes.

### Install an IDE for python

**Timing: 15 min**

Python programs can be composed as scripts using any text editor. However, we recommend using an Integrated Development Environment (IDE). There are several freely available IDEs for Python. The choice of IDE makes no difference the way Python programs run, but it may contribute to the coding experience, and visualization of outputs, such as tables and figures. All Python installations come with an IDE (*Idle*). Here we used *Spyder IDE,* an open-source and freely available program, for its user-friendly interface and advanced features.

5. Download the latest version of Spyder for your operating system from https://www.spyder-ide.org/.
  a. Install Spyder IDE following the operating system specific instructions.

***Note:*** If Python is installed through Anaconda platform, Spyder IDE can also be installed through Anaconda platform, which is listed in the applications on the anaconda-navigator.

## Key resources table

**Table:** 

REAGENT or RESOURCE	SOURCE	IDENTIFIER
**Deposited data**		

model.ode.pars	This paper	https://github.com/vehpi/T2D_str_pr/blob/main/model.ode.pars
bifurcation_data.dat	This paper	https://github.com/vehpi/T2D_str_pr/blob/main/bifurcation_data.dat

**Software and algorithms**		

Python3.10 or higher	Python Software Foundation	https://www.python.org/
Anaconda	Anaconda	https://www.anaconda.com/
model.ode	This paper	https://github.com/vehpi/T2D_str_pr/blob/main/model.ode
model_bif.ode	This paper	https://github.com/vehpi/T2D_str_pr/blob/main/model_bif.ode
xpp2python.py	This paper	https://github.com/vehpi/T2D_str_pr/blob/main/xpp2python.py
dynamics.py	This paper	https://github.com/vehpi/T2D_str_pr/blob/main/dynamics.py
model.py	This paper	https://github.com/vehpi/T2D_str_pr/blob/main/model.py
main.py	This paper	https://github.com/vehpi/T2D_str_pr/blob/main/main.py

**Other**		

Computer Hardware	N/A	N/A
T2D Progression Model	Yildirim et al.^1^	https://doi.org/10.1016/j.isci.2023.108324
animation.gif	This paper	https://github.com/vehpi/T2D_str_pr/blob/main/animation.gif

## Materials and equipment

In this section, we describe the hardware requirements to run the protocol. We also provide details about the computational model used. We briefly describe XPPAUT implementation of a dynamical model and explain the theoretical basis for continuation and bifurcation analysis.

### Computer hardware

The minimum hardware requirements for Python and XPPAUT are 4 GB of RAM and 5 GB of free disk space. Both Python and XPPAUT can be installed on MacOS, Windows and Linux machines.

### T2D progression model

The protocol is implemented using a recently published computational model that describes the dynamics of the obesity driven progression of type 2 diabetes (T2D) and remission through weight loss.^1^ Here we provide some background information and briefly describe the model.

**Figure 1:**
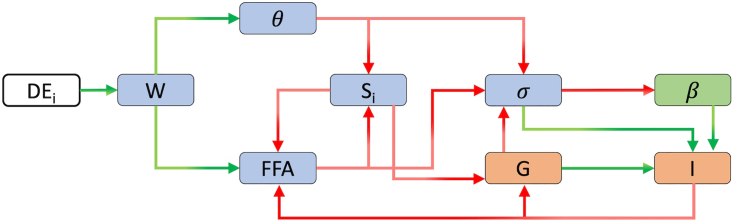
Model diagram DE_i_: Daily energy intake, W: Body weight, θ: Systemic inflammation, FFA: Plasma free fatty acids, *S*_*i*_: Insulin sensitivity, G: Plasma glucose, I: Plasma insulin, σ: β-cell function, β: β-cell mass. Green arrows indicate a positive/stimulatory relation, whereas red arrows indicate an inhibitory/negative relation. The timescales of the variables are color coded; green: slow variables, blue: intermediate timescale variables, orange: fast variables, white: parameters/constants.

In the model, T2D emerges as a result of insulin resistance in combination with insufficient insulin secretion, which is secondary to impaired β-cell function and reduced β-cell mass. β-cells secrete insulin in response to elevated plasma glucose levels, which induces glucose uptake in liver, muscle and adipose tissue cells. In the model, system dynamics are described by a set of coupled ordinary differential equations that determine the rates of change of body weight (body mass index, BMI), systemic inflammation level (θ), insulin sensitivity (*S*_*i*_), plasma free fatty acids (*FFA*), β-cell function (σ), β-cell mass (β), plasma glucose (G) and plasma insulin (I). The timescales of the model variables range from days to years and their interactions give rise to the emergence of insulin resistance and diabetes. The interactions between the model components are summarized in Figure 1. The details of the definitions of model variables and their mathematical formulations are described in Yildirim et al.^1^ In short, the system is at a healthy state at the baseline, where the total daily energy intake and expenditure are in balance, and a healthy BMI is preserved. A perpetual imbalance between energy intake and expenditure results in weight gain that initiates a chain of events that pushes the system out of the healthy state and eventually results in insulin resistance and diabetes. In the model, obesity is linked to insulin resistance and diabetes through increased FFA and inflammation levels, which reduce insulin sensitivity and β-cell function. Reduced β-cell function causes a decline in insulin levels. Together with reduced insulin sensitivity, reduced insulin levels lead to severe hyperglycemia. Prolonged hyperglycemia pushes β-cell metabolic rate to its limits and results in a further decline in β-cell function, and eventually results in β-cell death. In the model T2D emerges as a result of reduced insulin sensitivity and insufficient insulin secretion that results from reduced β-cell function and β-cell mass.

## Step-by-step method details

In this section we describe a step-by-step implementation of the protocol for analyzing the way metabolic system slowly progresses into the diabetic state as a result of increased body weight, and the way weight loss interventions result in diabetes remission. We start with explaining how to execute the model on XPPAUT and generate model simulations. We then use AUTO software to perform a numerical continuation analysis and generate bifurcation diagrams of the system. We then show how to export model and bifurcation diagram data into Python and identify the thresholds that separate healthy and diabetic states. We show how to use bifurcation diagram to calculate the calorie restriction necessary for successful remission, and how to predict the long-term behavior of the metabolic system under perturbations, such as weight gain and weight loss. We also introduce software packages for generating model simulations and animations.

### Executing the model on XPPAUT

**Timing: 15 min**

In this step, we provide details of how to execute the model on XPPAUT. We introduce the XPPAUT interface and necessary steps to run and simulate the model. For illustrations and figures, we use the *model.ode* script provided in KRT.

1. Open a terminal session and move to the directory where the XPPAUT is located.
  ***Note:*** If you have followed the online instructions, you should be able to start XPPAUT from the command line.
  a. On MacOS operating systems, type following by replacing the text in brackets by the path to the *model.ode* file;
    > xpp64mac [path to the model.ode]
    ***Note:*** For implementation details of computational models on XPPAUT using ode files please refer to the XPPAUT’s online documentation and provided examples at https://sites.pitt.edu/∼phase/bard/bardware/xpp/help/xppexample.html.
    **CRITICAL:** On MacOS machines, if XPPAUT executable file is copied to the *usr/local/bin* and it is on your path, you can start XPPAUT from any directory. On Apple silicon computers xppmacuniv should be used instead of xpp64mac. In fact, this binary works with both Apple and the Intel silicones, and it automatically detects the platform. On Linux machines, the instructions are the same as MacOS, whereas for Windows machines xpp should be used instead of xpp64mac in the command shown above. The XPP and AUTO interfaces are virtually identical on all operating systems, and following instructions apply to all operating systems.
    When the above command is run, X-window server will start automatically and XPP window in Figure 2 will pop-up.
    ***Note:*** The default background color and text size might be different on your platform. You may change the appearance of the XPPAUT either directly by declaring options within the ode file, or you can create a separate options script and call this script within the ode file. For details see online XPPAUT documentation.

**Figure 2:**
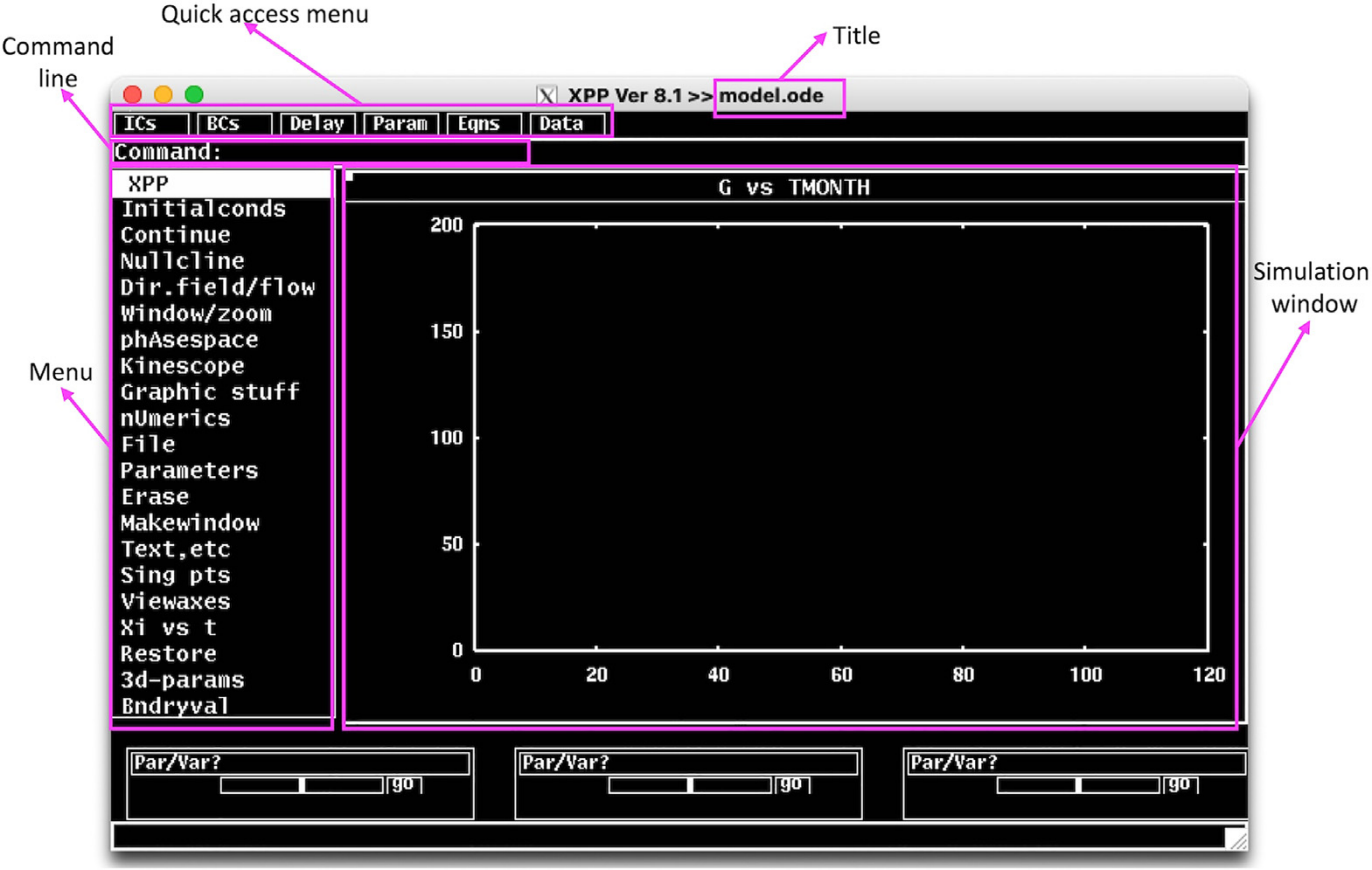
XPPAUT interface The screenshot of XPPAUT interface after model execution.
2. Major items on the XPPAUT’s main window are;
  a. **Title:** The version of XPPAUT and the name of the executed ode file.
  b. **Quick access menu:** A list of items that can be accessed quickly, which include initial conditions (ICs), boundary conditions (BCs), delays, parameter values (Param), equations (Eqns), and Data.
    ***Note:*** XPPAUT saves the time, dynamical variables and auxiliary variables data at each integration point.
  c. **Simulation window:** When model is integrated the solution trajectories appear in this window. Currently, the window shows time in moths (TMONTH) on the x-axis and plasma glucose (G) on the y-axis. This can be changed in Viewaxes in the XPP Menu.
  d. **XPP Menu:** Main XPP menu items. Menu items can be accessed by clicking on them or by keyboard shortcuts, where the capital letter in each menu item (or the letter inside the parentheses in sub menus) is the keyboard shortcut for the item.
    ***Note:*** Consecutive keyboard shortcuts can be used to access the menu>submenu items.
  e. A few important XPP Menu items for the execution of the protocol are;
    i. **Initialconds:** Provides access to the menu items to initiate the integration. To initiate the integration with current parameter values and initial conditions, follow Initialconds > (G)o, or press I > G on the keyboard. The following trajectory will emerge on the simulation window that shows the plasma glucose time course for 120 moths (Figure 3). Here we set the parameter *inc_i1 = 0.75* and *it1 = 300* in the Param quick access menu, to represent 75% increase at daily energy intake at T = 300 days. For a list of all model parameters and their explanations, please refer to Yildirim et al.^1^
      ***Note:*** The (R)ange option allows initiating multiple solutions from a range of initial conditions. (2)par range option allows generating multiple simulations for a range of parameter values. (L)ast option changes the initial conditions to the final point of the last simulation and integrates the model starting from this new initial condition. (M)ouse option allows initiating a new simulation from initial condition selected by a mouse click (m(I)ce option for multiple selections) on the simulation window. This is particularly useful when analyzing phase portraits.

**Figure 3:**
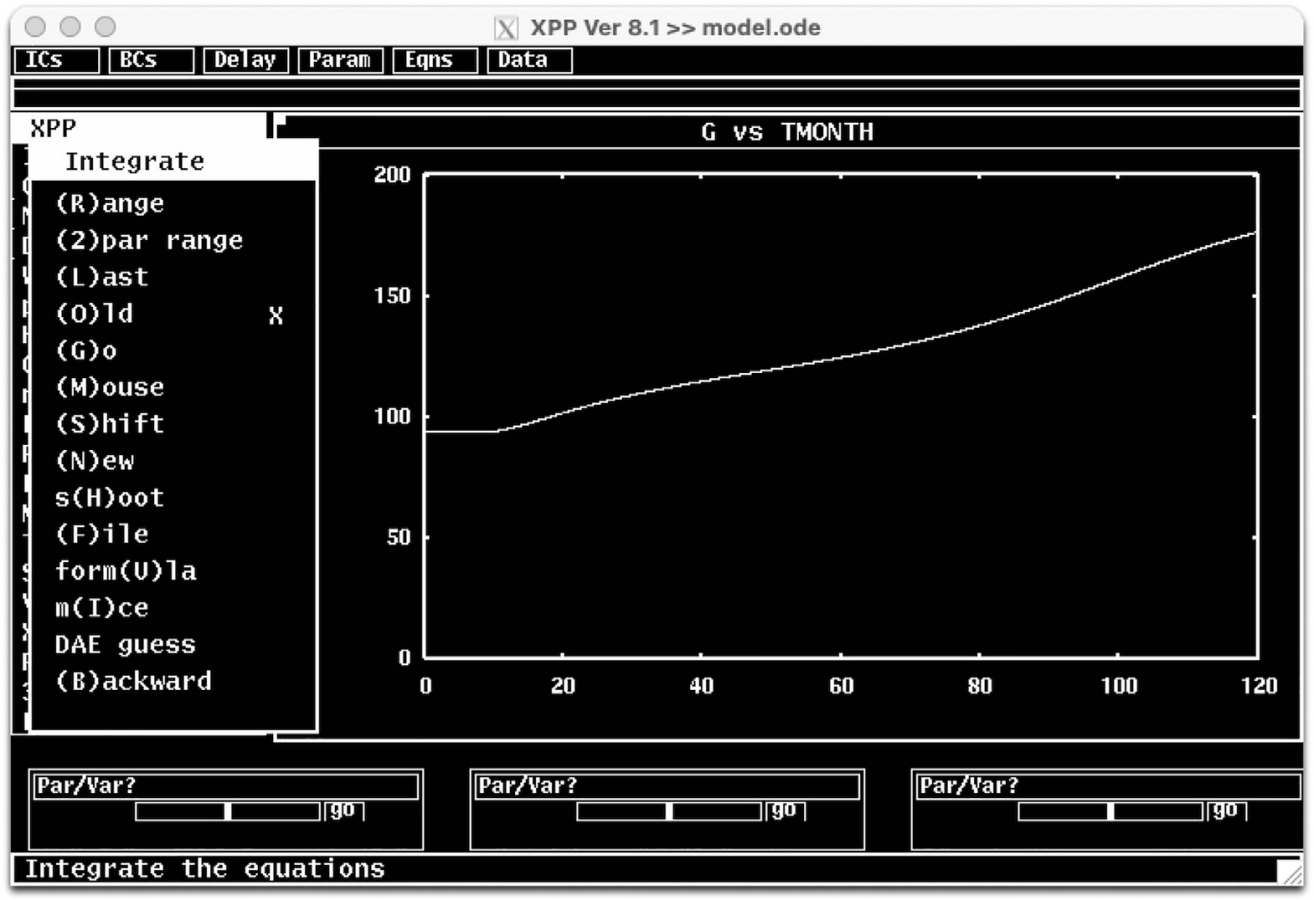
Initialconds submenu items and a representative model simulation for *inc_i1* = 0.75 and *it1* = 300 days
    ii. **Continue:** This option allows to continue simulation until an entered new time point. After choosing Continue (or pressing C), the new time point can be entered to the XPP command line.
    iii. **Nullcline:** Allows plotting and managing nullclines of the system on the phase plane.
    iv. **nUmerics:** Allows setting up numerical solver and associated parameters.
    v. **File:** This menu allows access to the main options such as changing *model.ode* file elements inside the XPPAUT interface (Edit), saving model information (Save info), and access to the AUTO interface (Auto).
    vi. **Makewindow**: Menu options for creating or destroying additional simulation windows (Figure 4).
      ***Note:*** The active window is indicated by a small square on the top left corner of the window. Any window can be made the active window by clicking anywhere on the window. The simulation on the active window can be refreshed by pressing R (Restore) and the active window can be cleared by pressing E (Erase).

**Figure 4:**
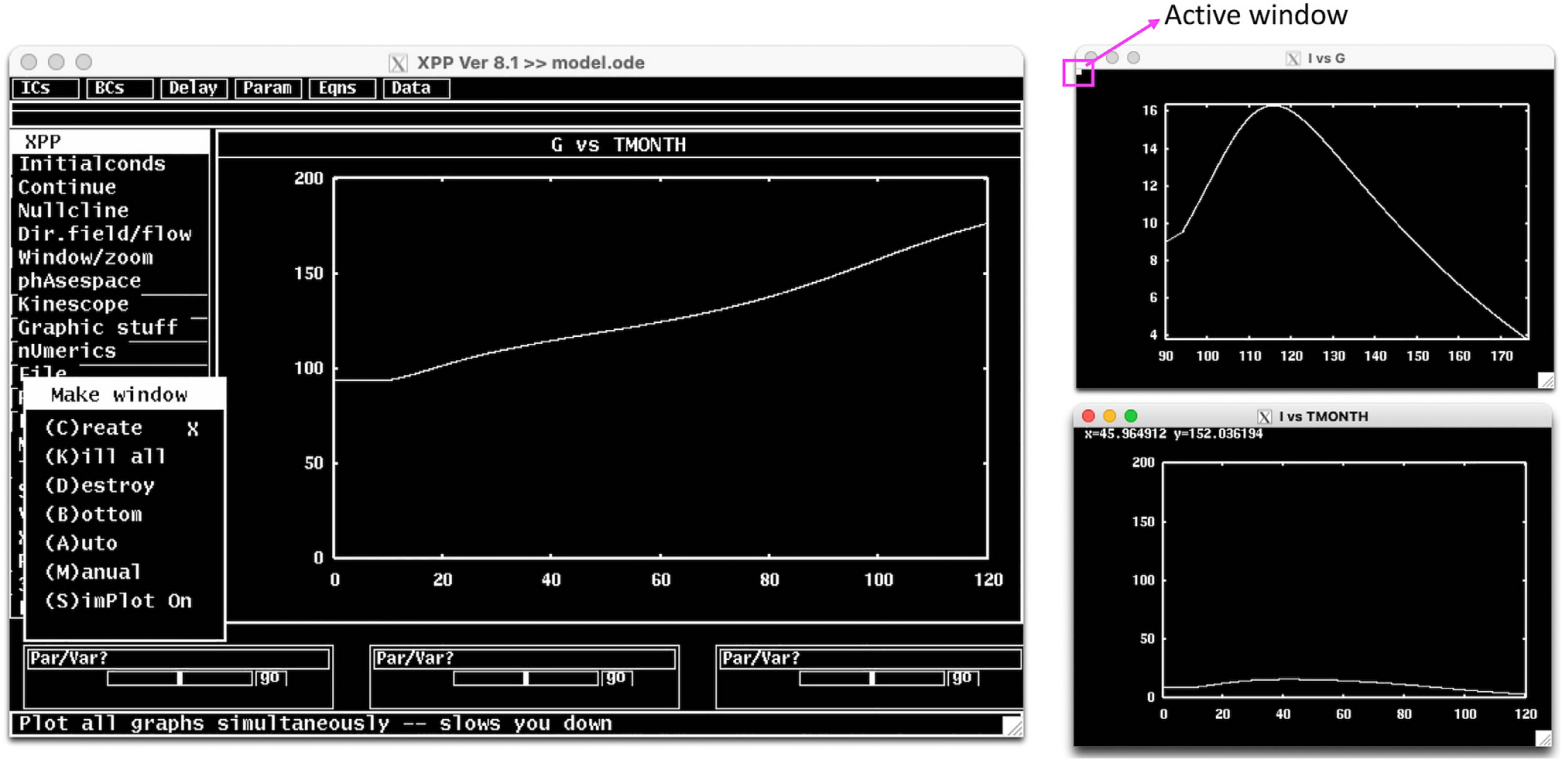
Additional XPP simulation windows can be created using Makewindow menu The currently active window is indicated by a small square on the top left corner of the window.
    vii. **Viewaxes:** This menu allows changing the active simulation window axes in 2D or 3D.
      ***Note:*** Although XPPAUT provides excellent tools for analyzing system’s dynamics, its visualization capabilities are limited. Therefore, we will use Python for generating model simulations and further analysis. This can be done manually, by creating a python implementation of the model. However, here we provide a package that contains a parser function written in Python to create a python script from the mode information exported from XPPAUT. The details of this function will be discussed at step 15, but for now we will describe how to save model information data on XPPAUT.
3. On the XPP Menu, go to File > Save info. This will save the model information as the *model.ode.pars* file.

***Note:*** The model information data file is provided in KRT (*model.ode.pars*).

### Using BMI as a parameter for the continuation analysis

**Timing: 30 min**

In this step, we describe how to use *BMI* (a dynamically changing model variable) as a parameter to perform a continuation analysis. This way the effect of weight gain on the emergence dynamics of type 2 diabetes can be observed through a bifurcation diagram. This requires creating a new model, where BMI is defined as a model parameter that is used to calculate body weight. For a detailed description of bifurcation analysis and numerical continuation, please refer to the (Guckenheimer, 1980).^4^

***Note:*** Here we use BMI instead of body weight, since BMI provides more information about the obesity status of the subject regardless of height.

***Note:*** In the model depicted in Figure 1, variables have fast, intermediate and slow timescales. *G* and *I* are the fast variables that act on the order of hours-days; *FFA*, *S*_*i*_, *θ*, *σ*, and *BMI* are intermediate timescale variables that act on the order of weeks-months; and *β* is a slow variable that acts on the order of years. The timescales of the variables determine the way they dynamically respond to perturbations, such as a change in daily energy intake (*DE*_*i*_). Since we are interested in long-term slow dynamics of the system in relation to weight gain, we focus on the slow variable β and the way its steady state value changes in response to the changes in body weight or BMI.

4. Create a new copy of *model.ode* file and name it *model_bif.ode*.
  a. Open *model_bif.ode* with a text editor, and define *BMI* as a parameter of the system.
  b. To represent W with body mass index (*BMI*), define following relation in the model;

(Equation 1) $$W=BMI\cdot h^{2}$$

Where *h* is the height in meters, and it is a parameter of the model. This reduces the number of variables in the model by one.

5. Save *model_bif.ode* into the directory, where other protocol files are located.

***Note:*** A copy of the *model_bif.ode* file is included in KRT.

**CRITICAL:** The model presented in *model_bif.ode* should only be used for generating the bifurcation diagrams of the system using *BMI* as the bifurcation parameter, and not for model simulations. Because the model presented in *model_bif.ode* does not include body weight (*W*) or *BMI* as a dynamic variable.

### Generating the bifurcation diagram using AUTO

**Timing: 30 min**

In this step, we describe how to start AUTO, and generate a bifurcation diagram of the system using *model_bif.ode* file and BMI as the bifurcation parameter. We will explain important numerical parameters, and propose solutions to potential problems.

**Figure 5:**
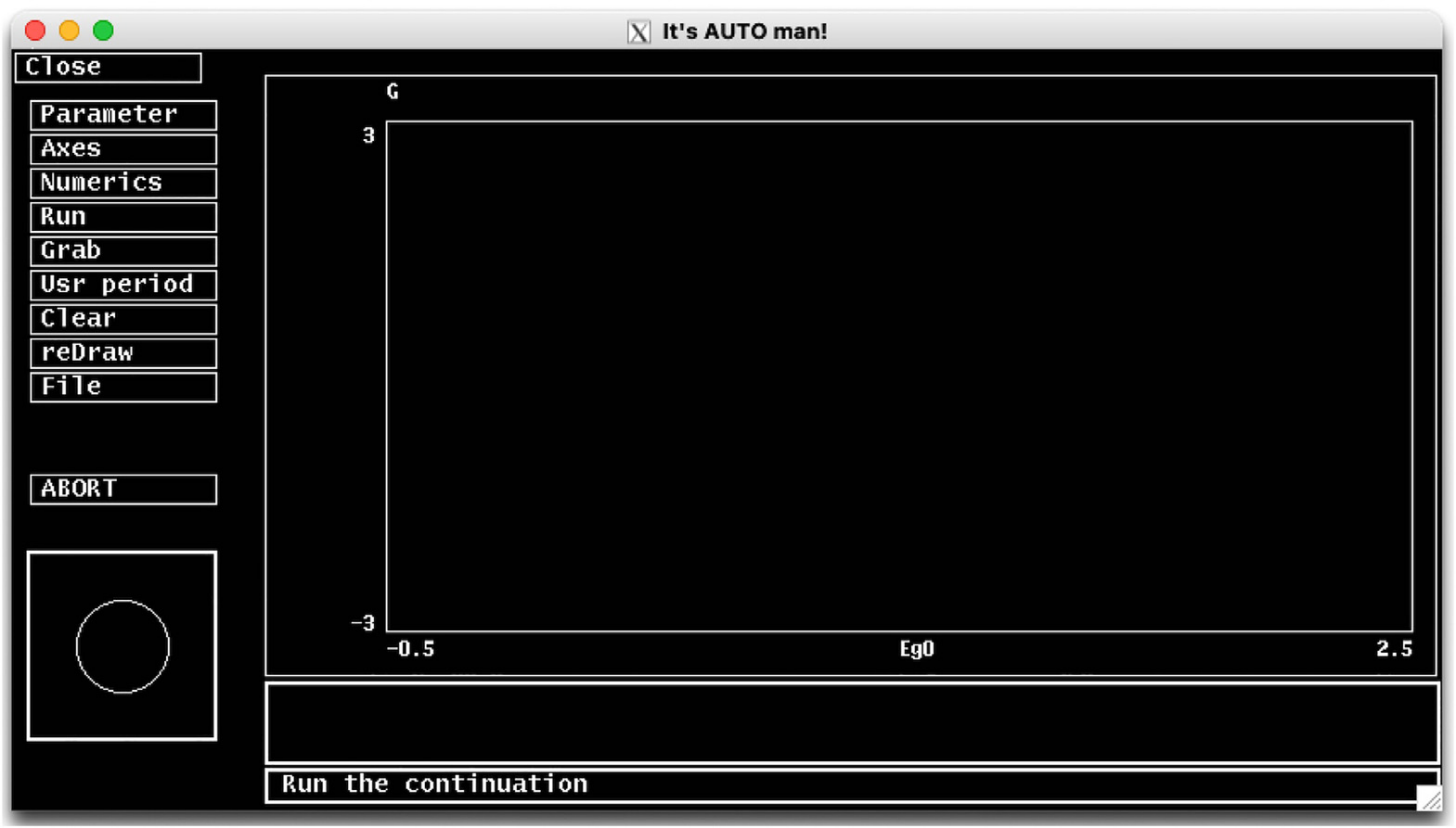
The AUTO interface
  The AUTO menu items are located on the left side of the main simulation window. Important AUTO menu items for the protocol are as follows.
  a. **Parameter:** On this menu you can specify the bifurcation parameter, which is the first parameter on the list. You should type the name of the parameter as it is declared in the ode file. AUTO defaults to the first parameter defined on the ode file (*Eg0* in this case).
  b. **Axes:** On this menu, you can set the variable you want to draw on the y-axis. In other words, the major variable of interest. On this menu, you can also set the axes ranges. By default, AUTO sets the y-axis to the first dynamic variable on the ode file (*G* in this case).
  c. **Numerics:** On this meu you can set the numeric parameters for running continuation analysis.
  d. **Run:** This initiates the continuation calculations from the current steady-state solution.
  e. **Grab:** Selecting this option allows grabbing a point on the diagram to continue to track the solutions.
    **CRITICAL:** If the grabbed point is a bifurcation point, this allows you to select on which branch the numerical solver to continue. For instance, when a Hopf-Bifurcation point is grabbed, you can tell AUTO to keep track of the branch of the steady state solutions or the branch of the periodic solutions.
  f. **Clear** and **reDraw** options allow clearing the screen and redrawing the latest diagram, respectively.
    ***Note:*** Clear does not erase the bifurcation diagram information, it only clears the simulation window. To reset the bifurcation diagram, you must choose File > Reset diagram option.
  g. **File:** File menu provides options to save the diagram information as an exportable dat file, printing diagram as a post-script file, loading a new diagram from file, clearing current grabbed point, or resetting the diagram.
8. Go to Parameter menu on AUTO and set the first parameter on the list to bmi. Click Ok or press the tab button on the keyboard to save.

***Note:*** The rest of the parameters on the params menu are irrelevant for our analysis.

6. Execute the *model_bif.ode* file on XPPAUT as described above.
7. On the XPP menu, go to File and select Auto to start the AUTO interface (Figure 5).
  ***Note:*** When AUTO is initiated, the XPP window remains open and active, where any changes made in XPP affects the AUTO.

9. Go to Axes menu and select hI-lo option to setup the axes on the main simulation screen on AUTO. The hI-l0 menu items should be set as follows;
  a. **Y-axis:** The main variable of interest to be plotted on the y-axis. Set this to B (β-cell mass in the model).
  b. **Main Parm:** The bifurcation parameter. Set this to bmi.
  c. Set axes limits to **Xmin:**25**, Xmax:**50**, Ymin =** 0 **,Ymax =** 2000.
    **CRITICAL:** On the Axes>hI-lo menu, the Secnd Parm option can be used for generating a two-parameter continuation curve to investigate how a bifurcation point changes with respect to a second parameter.
  d. Click Ok or press tab to save the changes.
10. Go to Numerics menu to setup the numerical parameters for continuation analysis. Important items on the Numerics menu are listed below. Set their values to the values given in brackets and leave the rest of the parameters at their default values.
  a. **Nmax[8000]:** The maximum number of steps taken along any branch, while finding steady states. The AUTO stops after this many steps are taken. If AUTO prematurely stops due to reaching Nmax, problem 2 in troubleshooting section should be implemented.
  b. **Npr[500]:** After each Npr steps, AUTO provides complete information about the process. Setting Npr to a large number may speed up the process.
  c. **Ds[0.02]**: Initial step size of the bifurcation parameter for the continuation analysis. AUTO uses an adaptive variable step size. Therefore, the magnitude of Ds is not very important. However, the sign of Ds is very important, and it tells the AUTO the initial direction to change the bifurcation parameter.
  d. **Dsmin[0.001]**: The magnitude of minimum step size.
  e. **Dsmax[0.5]:** The magnitude of allowed maximum step size.
    **CRITICAL:** When choosing a suitable Dsmax, the scale of the bifurcation parameter should be taken into account. If Dsmax is set to a large value, AUTO might miss some important points. So, if it seems to be missing a stability transition, or if the diagram is jagged, reducing this number might help.
  f. **Par Min[25]:** This is the minimum value for the bifurcation parameter. The calculation stops if the parameter value falls below his value.
  g. **Par Max[50]:** This is the maximum value for the bifurcation parameter. The calculation stops if the parameter value increases above this value.
  h. **Norm Max[1e+6]** The upper bound for the L2 norm of the solution. If the L2 norm of the solution is greater than this number, the calculation will stop. For large models with variables on large scales, Norm Max should be set to a large value.
  i. Click Ok or press tab to save the changes.
    ***Note:*** These internal AUTO options can be directly declared within the ode file using the code block shown below.
    # AUTO options
    @ NTST=20, NMAX=8000,NPR=500,PARMIN=25,PARMAX=50,NORMMAX=1e6
    @ AUTOXMIN=25,AUTOXMAX=50,AUTOYMIN=0,AUTOYMAX=2000,AUTOVAR=b
    @ DSMIN=.001,DSMAX=0.5,DS=.02
11. Now the numeric parameters are all set and continuation analysis can be initiated.
  **CRITICAL:** Continuation calculations are initiated from the current initial condition, which is set on the XPP. Therefore, the current initial condition on XPP should be a steady state solution of the system. If the current initial condition of the model is not set to a steady state on XPP, AUTO may fail to converge. Please see the problem 1 and proposed solution in troubleshooting section.
  a. Press the Run button on AUTO Menu, and a submenu will appear (Figure 6, top left).

**Figure 6:**
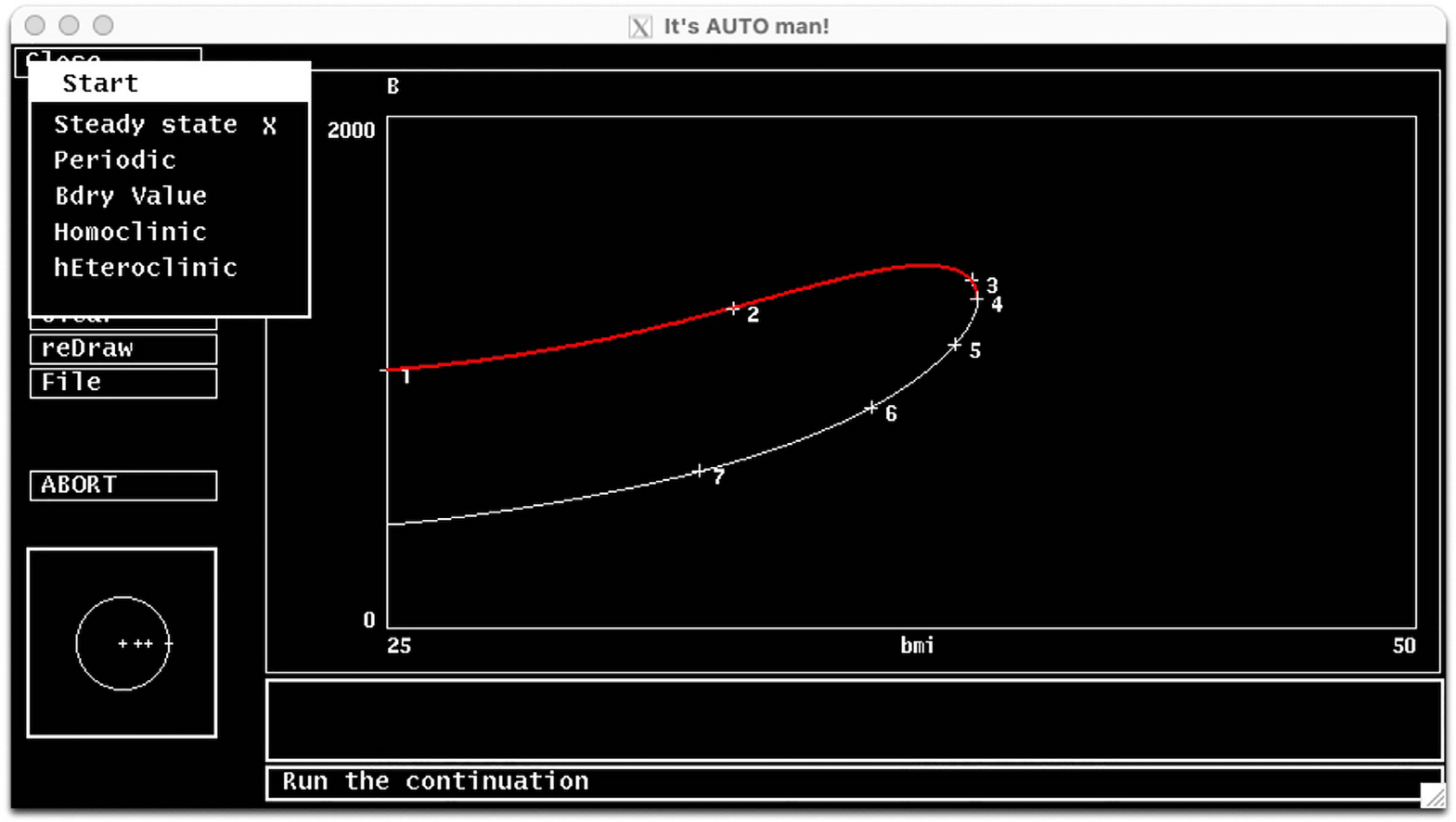
First segment of the bifurcation diagram on AUTO Upper stable (red) and unstable (white/black) branches of the bifurcation diagram.
  b. Select the ‘Steady state’ option on that submenu for auto to start.
  c. A steady state solution curve should appear on the AUTO’s simulation screen (Figure 6).
    **CRITICAL:** If AUTO reaches to the maximum number of steps specified by Nmax in Numerics menu before going through the entire parameter range, the continuation calculations will stop prematurely. In that case, you should implement the solution proposed for problem 2 in troubleshooting section.
    **CRITICAL:** AUTO keeps track of the steady state solutions within the parameter range specified as [Par Min, Par Max] in the Numerics menu. However, the system might have other steady states, which are represented by a different curve within the allowed parameter range. Indeed, setting bmi = 50 and running simulations on XPP reveals that solutions converge to a lower B stable steady state indicating the existence of a lower branch of stable steady states.
12. Generate the lower branch of stable steady states and add it to the current diagram. To do this;
  a. Go to the XPP window, and switch the y-axis on the main simulation screen to B using Viewaxes menu, and set the y-axis limits to 0 and 2000.
  b. Press P on the keyboard to allow parameter input. Type in bmi and press enter. Now type in 50 and hit the Enter to set the value of bmi to 50, which is a high level.
    ***Note:*** You may also change multiple parameter values at once by click on Param on the Quick Access Menu on XPP, which lists all parameters and their values.
  c. Run the simulation by choosing Initialcond > (G)o (or press I > G on the keyboard). You should see that B asymptotically converges to a low stable steady state.
  d. Then repeat the process using the last point from previous simulation as the new initial condition by choosing Initialcond > (L)ast (or press I > L on keyboard). Since AUTO works best when initiated close to a steady state, repeat this step for a few times until simulation results in a horizontal line indicating that the system has reached to an equilibrium.
  e. Go to AUTO and open Numerics menu. Since bmi is now set to 50, you should tell AUTO to change bmi (the bifurcation parameter) in the negative direction by making Ds negative (‒0.02). Click Ok or press tab to save the changes.
    **CRITICAL:** If **grab** function in AUTO has been used in the previous steps, you need to clear the grabbed point first before you continue. In that case, refer to the problem 3 and proposed solution in troubleshooting section.
13. Click Run, and a warning message will show up asking if you want to destroy the current diagram.
  a. Choose ‘No’ to keep the information from previous simulation available. You should see a new stable branch emerging from the lower right and extending to the left (Figure 7, complete bifurcation diagram).

**Figure 7:**
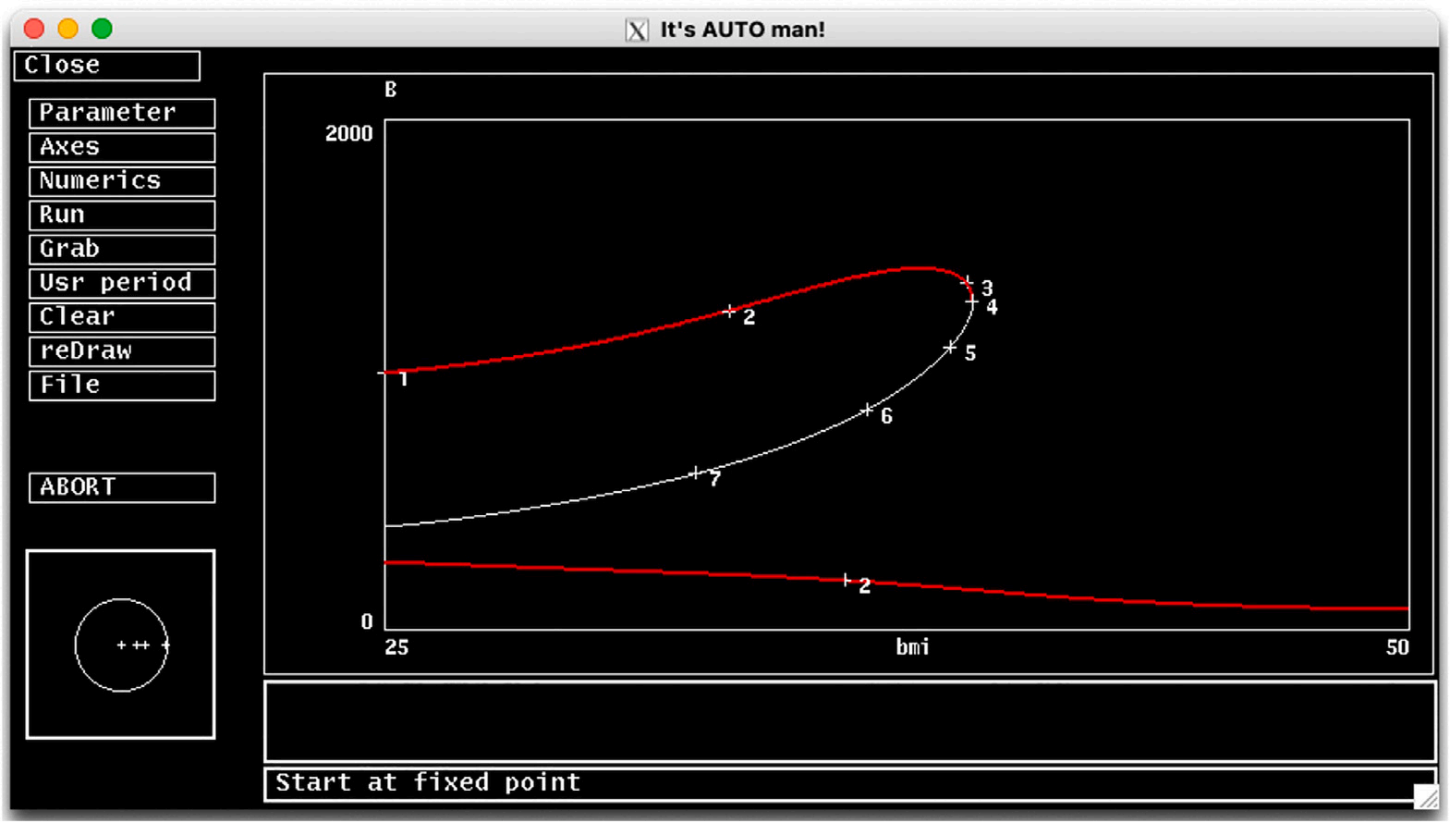
The bifurcation diagram Stable steady state solutions (red) and unstable steady state solutions (white/black) of the system.
14. Go to File menu on AUTO and select All Info to save the bifurcation diagram data in a *dat* file.

***Note:*** This data will be used to continue the analysis on Python, which provides more flexibility and better visualization options. We will explain the details of the diagram in step 16. A *dat* file of the bifurcation diagram data is provided in KRT as *bifurcation_data.dat.*

### Import model data to python

**Timing: 15 min**

This section describes how to import model data into Python. Importing model and bifurcation diagram data into Python allows using Python’s more advanced and versatile features for simulation and analysis.

**Figure 8:**
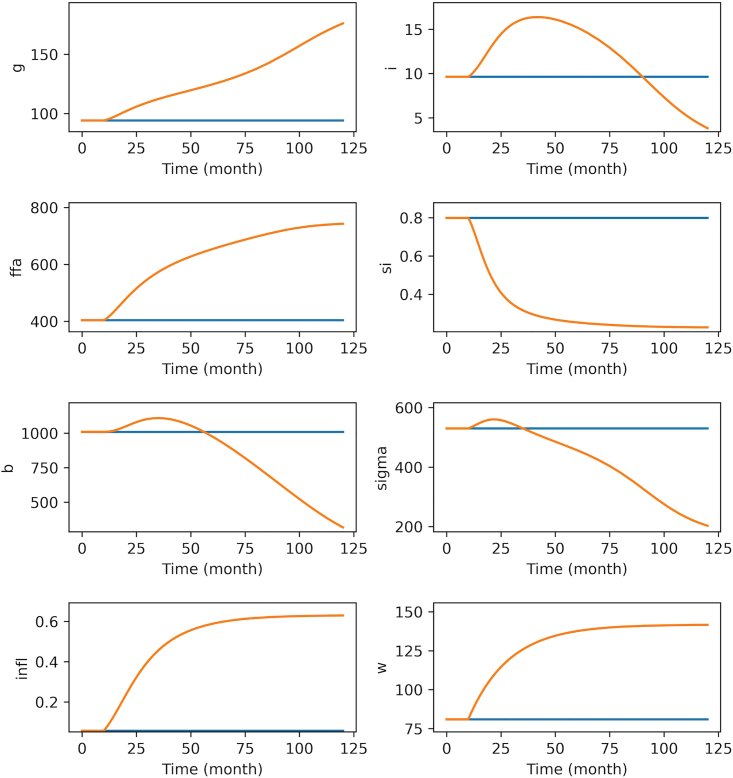
Model simulations generated using the simul function For blue simulations default parameters were used. For orange simulations inc_i1 is increased to 0.75 and it1 is set to 300 to simulate the effect of increasing daily energy intake by 75% at T = 300 days (10 months). The second set of simulations are superimposed on the existing figure by setting keep_old = True and fign to the current figure number.

15. Open Python IDE.
  ***Note:*** On all operating systems, Spyder can be started from Anaconda-Navigator or by running Spyder command on a Terminal session. If you do not prefer using an IDE, you can use any python console to run the protocol on all operating systems. You may also use an interactive Python notebook such as Jupyter Notebook.
  a. Move to the folder that contains *model.ode.pars* file you saved at step 3a and *bifurcation_data.dat* file saved at step 14.
  b. Make sure *xpp2python.py* and *dynamics.py* files (see key resources table) are also in this directory.
  c. Use following command to import functions stored in *xpp2python.py* module into the workspace.
    > from xpp2python import ∗
    This module includes 3 functions.
    i. **read_info:** This function takes the name of the file that contains the model information exported from XPPAUT (*model.ode.pars*) as input. The function parses the information saved in the file, and returns this information as a python dictionary object. This information is used to create a python script for the model in the next step.
    ii. **create_script:** This function uses model information exported from XPPAUT to create a python script for the model.
    iii. **simul**: This function integrates the model and generates plots.
  d. Use following command to create a python executable script for the model.
    > info=create_script(‘model.ode.pars’)
    ***Note:*** This will create a python script with the name *model.py* and save it to the current working directory. The script contains a function (odde) that defines the model equations, parameter values, and initial conditions as exported from the XPPAUT. This function call also saves the model information, such as variable names, parameter names, initial conditions, etc. into the info dictionary object.
    ***Note:*** Model information can also directly be stored into the info dictionary object by using read_info function;
    > info=read_info(‘model.ode.pars’)
  e. Run following command to import the model to the workspace.
    > from model import ∗
    ***Note:*** This will import the model ODE function (odde), parameters (pars), initial conditions (y0), and simulation time span (t0 and tend) into the workspace.
  f. Confirm the model by calling the simul function;
    > sol=simul(odefun=odde, tspan=[t0,tend], y0=y0, pars=pars,info=info, fign=None, keep_old=False)
    ***Note:*** This will integrate the model and save the solution information into the sol object. If you want to see the model simulations, you can set fign to a positive integer for python to create a figure with number fign (Figure 8, blue).
    **CRITICAL:** The create_script function translates user defined XPP functions into Python *lambda* functions. If Python raises an ‘*undefined function or variable’* error associated with one of the user defined functions in XPP, please use the solution suggested for problem 4 in troubleshooting section.
    ***Note:*** In the simul function call, you may set the keep_old option to True and set fign to an existing figure number to superimpose the new set of simulations on an existing set(s) of simulations for comparison (Figure 8, orange). The figure number can be found on the top of the Python figure window.

### Generate a bifurcation diagram

**Timing: 15 min**

This section describes how to plot the bifurcation diagram on Python and summarize the dynamic behavior of the system. We also show how the branch of unstable steady states serves as a threshold that separates the healthy state from the diabetic state.

**Figure 9:**
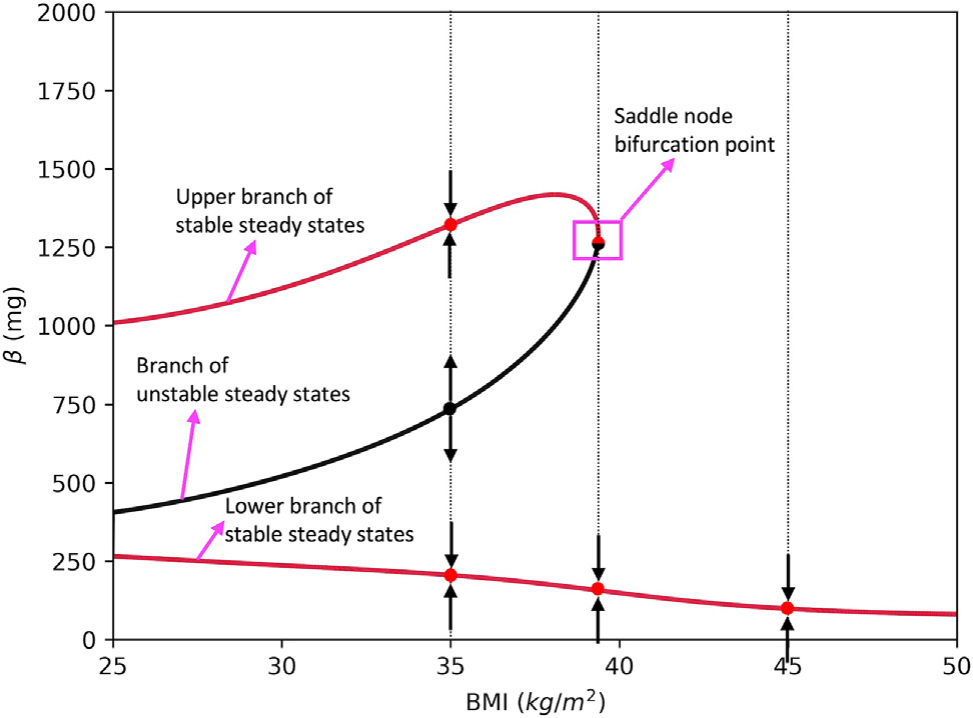
The bifurcation diagram of the system projected onto BMI-β axes Red curves show the stable steady states of β, whereas black curve shows the unstable steady states of β for corresponding BMI value. Vertical dotted lines show BMI = 35, 39 and 45 cross sections, where red dots show stable steady states, and black dot shows unstable steady state. The black arrows show the way solutions behave near each steady state. The vertical lines, arrows and text do not appear on the figure automatically, and they are added later for illustration purposes.

16. Use following command to import the functions contained in the *dynamics.py* module.
  > from dynamics import ∗
  This module contains functions for model simulation and analysis.
  a. Use bif_fig function to create a bifurcation diagram using the bifurcation diagram data stored in *bifurcation_data.dat* and current parameter set.
    > bif_fig(bif_name=’bifurcation_data.dat’, pars=pars)
    ***Note:***Figure 9 shows that for each fixed BMI value between 25‒39, the system has 3 steady state solutions; two stable and one unstable steady states. System goes through a saddle node bifurcation (SNB) at BMI≈39 kg/m^2^, where the upper stable steady state coalesces with the unstable steady state and disappears. Hence, for BMI>39, there is a single stable steady state. This diagram shows that for BMI values above 39 kg/m^2^, all solutions converge to the corresponding lower β stable steady state. However, for BMI between 25‒39 kg/m^2^ solutions either converge to a lower or higher β steady state depending on which side of the unstable steady state the solution was initiated. Therefore, for BMI values between 25‒39 kg/m^2^ branch of unstable steady states separates the phase plane and serves as a threshold.

### Explore emergence dynamics of diabetes and remission

**Timing: 15 min**

The model behaves as described above for fixed BMI. However, in the model, BMI is a dynamic variable that changes over time. In the model, an increase in *DE*_*i*_ results in weight gain, whereas a decline in *DE*_*i*_ represents a calorie restriction or a diet intervention that results in weight loss. We now show how to use bifurcation diagram to capture the association between level of BMI and β for the emergence of T2D. We also show how to calculate the level of calorie restriction necessary for successful remission at each region on the BMI-β plane.

**Figure 10:**
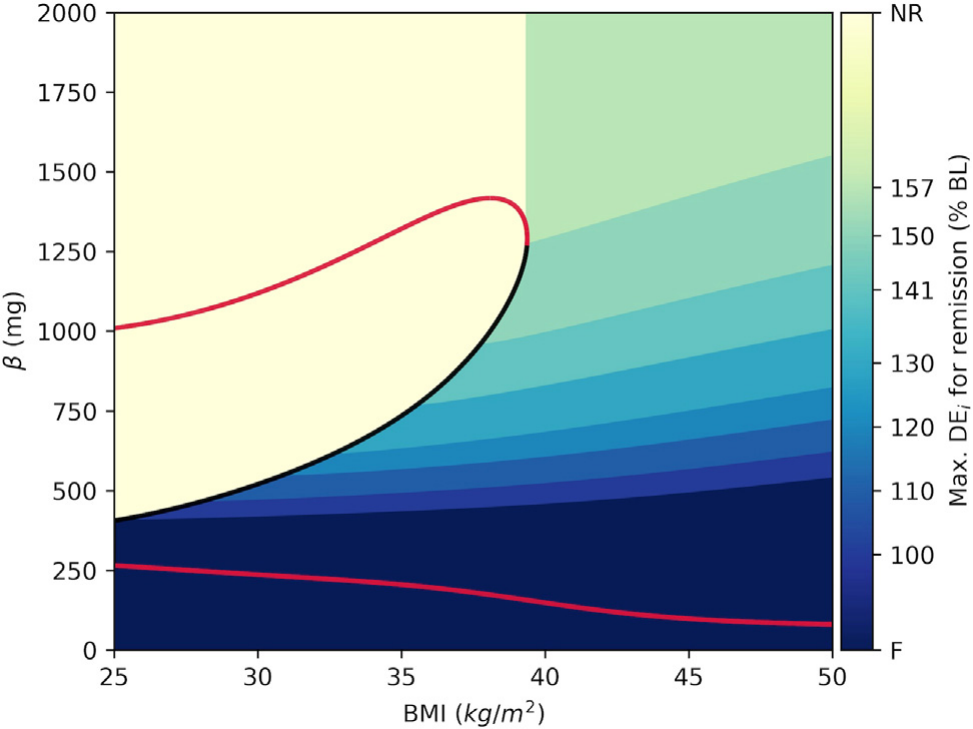
Bifurcation diagram with calorie restriction boundaries for successful remission The color bar shows the maximum *DE*_*i*_ levels relative to baseline (percentage of BL) necessary for successful remission within the corresponding region. NR: no restriction; F: failure.

17. Set dec_bound option to True in bif_fig function to create a bifurcation diagram with a color map that shows the necessary level of calorie restriction for remission for each BMI and β value (Figure 10).

> bif_fig(bif_name=’bifurcation_data.dat’, pars=pars,dec_bound=True)

***Note:*** In Figure 10, the color map shows the levels of BMI, where T2D emerges due to a decline in β. The color map also shows the level of calorie restriction necessary for successful remission in each region. In the yellow region labeled as no restriction (NR) the solution trajectories are attracted by the high β stable steady state and hence T2D does not emerge. However, in the region marked by the shades of green to blue, T2D emerges due to the decline in β. Shades of green and blue also show the level of the calorie restriction necessary for remission in each region, which is calculated as the maximum DE_i_ over the baseline necessary for successful remission in the region.

***Note:*** Setting dec_bound = True in the bif_fig function calculates the calorie restriction boundaries for successful remission. For this, the algorithm generates a grid of initial conditions for BMI = 50 and β values in the [0,2000] range (*β*_*0,i*_). For each initial condition, algorithm generates model solutions for a range of DEi values shown in the color bar between [175,100] percent of the baseline (BL). Afterwards, the algorithm determines the boundaries for each calorie restriction region as the trajectory that is initiated from the lowest β value that reaches to the threshold with corresponding DEi restriction. Please see the bif_fig function’s code in *dynamics.py* module and the remarks therein for the details of the algorithm.

**CRITICAL:** Repeating the analysis for different sets of parameters is possible, but a new bifurcation diagram also needs to be generated for the new parameter set on XPPAUT following steps 6 through 17.

**Figure 11:**
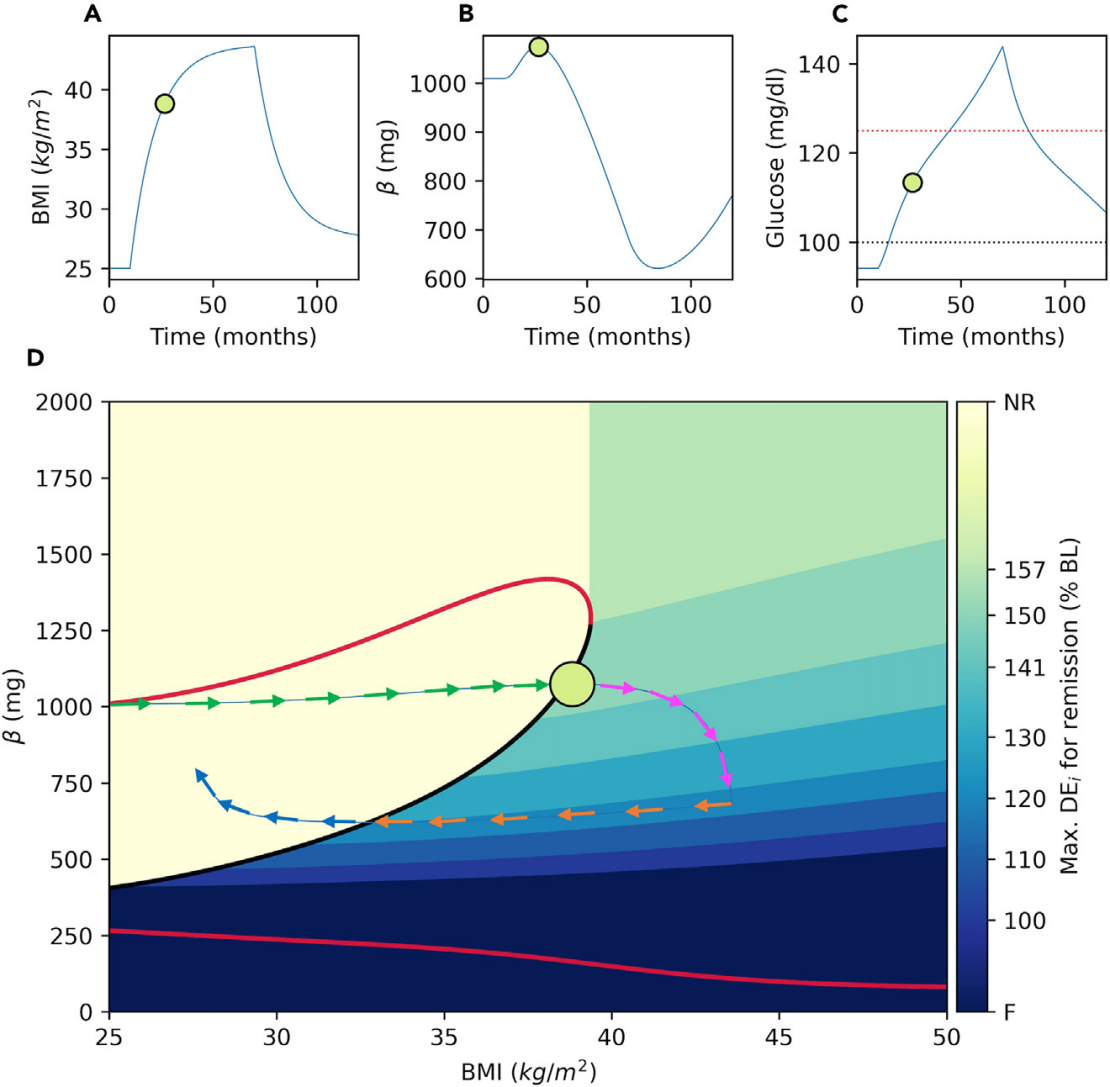
A snapshot of the animation created using the animation() function BMI (A), β (B) and Glucose (C) time courses are shown. (D) Solution trajectory (blue) is superimposed onto the bifurcation diagram. The DE_i_ is increased by 175% over the baseline (BL) at T = 10 months and set back to 110% of the BL at T = 70 months. In animations, the filled circle represents the phase point and it navigates through the trajectories. The color of circle is mapped to the glucose level shown on the color bar as an indicator of diabetes status. The arrows show the pre-diabetes (green), diabetes emergence (magenta), weight loss (orange) and remission (blue) phases. In panel D, the size of the circle is proportional to BMI.

18. Use animation function to create dynamic animations that show the way the phase point moves through the phase space. A GIF file of a representative animation is provided in KRT (*animation.gif*), where Figure 11 shows a snapshot of the animation.

> ani=animation()

***Note:*** The trajectory is initiated from (BMI = 25, β = 1000), where DE_i_ is increased by 75% over the baseline (BL) at T = 10 months. Initially, the phase point moves right and upward (Figure 11, green arrows). In the yellow region, BMI is increased due to the positive imbalance between energy intake and expenditure, meanwhile β is increased since the phase point is in the basin of attraction of the high β stable steady states. When the phase point passes through the threshold, the trajectory switches direction in the β-axis by entering to the basin of attraction of the lower β stable steady state. In this region the phase point keeps moving to the right, but it starts moving downward (Figure 11, magenta arrows). Inside the region marked as DE_i_ = 150% BL, DE_i_ must be reduced from 175% BL to a value less than or equal to the 150% of the BL for successful remission. Over time the phase point keeps moving downward towards darker regions, which implies a stricter calorie restriction for remission. At T = 70 months, the trajectory takes a sharp left turn indicating a diet intervention imposed by setting DE_i_ to 110% of the BL (Figure 11, orange arrows). Since the phase point is inside the region marked by DE_i_ = 120% BL, this calorie restriction is able to move the phase point to the left side of the threshold, where it starts moving upward in the β-axis (Figure 11, blue arrows). Notice that the trajectory moves left and downward after the intervention, because decline in β continues until phase point reaches to the threshold. This implies the importance of the pace of the weight loss, where weight loss on a slower pace would not be able push the phase point beyond the threshold and remission would fail.

***Note:*** The animation function can also be used to create custom animations for different scenarios by changing the default values of its parameters;

> ani=animation(fignum=7,it1=300,it2=2100,inc_i1=0.75,inc_i2=0.1, t0=0,tend=3600, dec_bound=False)

Where, fignum is the figure number, it1 and it2 are the time points where changes in daily energy intake inc_i1 and inc_i2 are initiated, respectively. The parameters, t0 and tend determines simulation start and end times in days, respectively. Setting dec_bound = True plots the color map that shows the necessary level of calorie restriction for remission for each BMI and β value as shown in Figure 11.

***Note:*** The animation function returns a matplotlib.animation.FuncAnimation object (ani), which can be saved in gif file format using following command;

> ani.save("ani.gif", dpi=200,writer=PillowWriter(fps=25))

**CRITICAL:** Protocol can also be run on interactive Python notebooks such as Jupyter Notebook. On these notebooks, animations may not run inline. In this case, refer to the problem 5 and suggested solution in troubleshooting section.

## Expected outcomes

Figures 9, 10, and 11 show that for each BMI value between 25‒39 kg/m^2^, branch of unstable steady states separates the phase plane and serves as a threshold. On the right side of (or below) the threshold, solutions converge to the lower β stable steady state and T2D emerges. On the other hand, on the left side of (or above) the threshold, solutions converge to a higher β stable steady state. In this section, we use the protocol to analyze the model simulations under different weight gain and weight loss scenarios. We show how to use the protocol to analyze the long-term dynamic behavior of the system.

**Figure 12:**
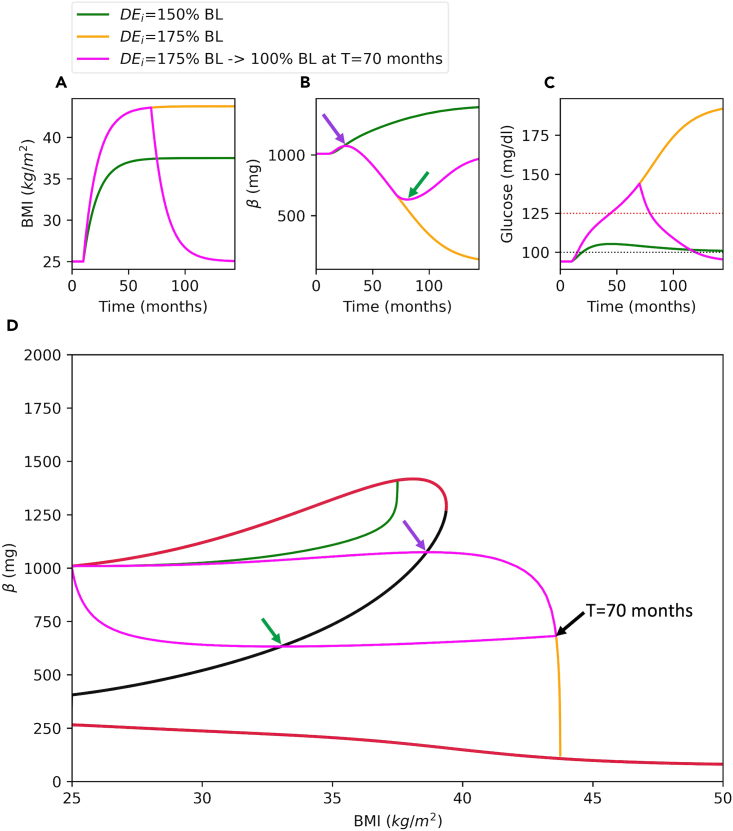
Solution trajectories against the bifurcation diagram BMI (A), β (B) and Glucose (C) time courses are shown for different parameter sets. (D) Solution trajectories are superimposed onto the bifurcation diagram. For green simulation, DE_i_ is increased by 50% over the baseline (BL) at T = 10 months. For orange simulation, DE_i_ is increased by 75% at T = 10 months. For magenta simulation, DE_i_ is increased by 75% at T = 10 months and set back to the baseline at T = 70 months to simulate the effect of a calorie restriction. Purple and green arrows in panels B and D show the peak and nadir of green trajectory from a different perspective, respectively. In panel C, black and red horizontal dotted lines mark the glucose cut offs for emergence of prediabetes (100 mg/dL) and T2D (125 mg/dL).

Running outputs function with fn = 12 option crates a figure with 3 solution trajectories superimposed onto the bifurcation diagram (Figure 12).

> outputs(fn=12)

Figure 12 shows the bifurcation diagram along with superimposed solution trajectories. Green trajectory remains on the left-hand side of the threshold and converges to a high β stable steady state (Figure 12D). Therefore, diabetes does not emerge at all, where glucose levels always remain below diabetes cut off (Figure 12C, green). However, the orange trajectory passes through the threshold and converges to a low β stable steady state (Figure 12D, orange), which results in high glucose and diabetes (Figure 12C, orange). On the other hand, magenta trajectory passes to the right hand-side of the threshold, where it starts moving downward towards a lower β stable steady state. In magenta simulations, diabetes also emerges as indicated by high glucose levels (Figure 12C, magenta). However, at T = 70 months, the trajectory makes a sharp left turn due to the induced calorie restriction (Figure 12D, magenta). The trajectory moves to left and downward until it reaches to the threshold since it is in the basin of attraction of the lower β stable steady state. Once the trajectory passes to the left side of the threshold, it enters to the basin of attraction of the high β stable steady state and starts moving upward indicating a successful remission evident from the declined glucose levels (Figure 12C, magenta). The magenta simulation takes it maximum (Figures 12B and 12D, purple arrow) and minimum (Figures 12B and 12D, green arrow) values in β as the trajectory passes through the threshold. Because, at these points the direction of the flow switches in β axis (Figure 12D).

Figure 12D shows that trajectories need to pass through the threshold to switch direction in the β-axis. Once the trajectory passes to the right side of the threshold, diabetes emerges on the time-scale of β. Therefore, remission can only take place if a weight loss intervention pushes the phase point back to the left side of the threshold. This depends on the time spent on the right side of the threshold and the intensity of the calorie restriction. We will now show how the level of calorie restriction is related to the success remission.

**Figure 13:**
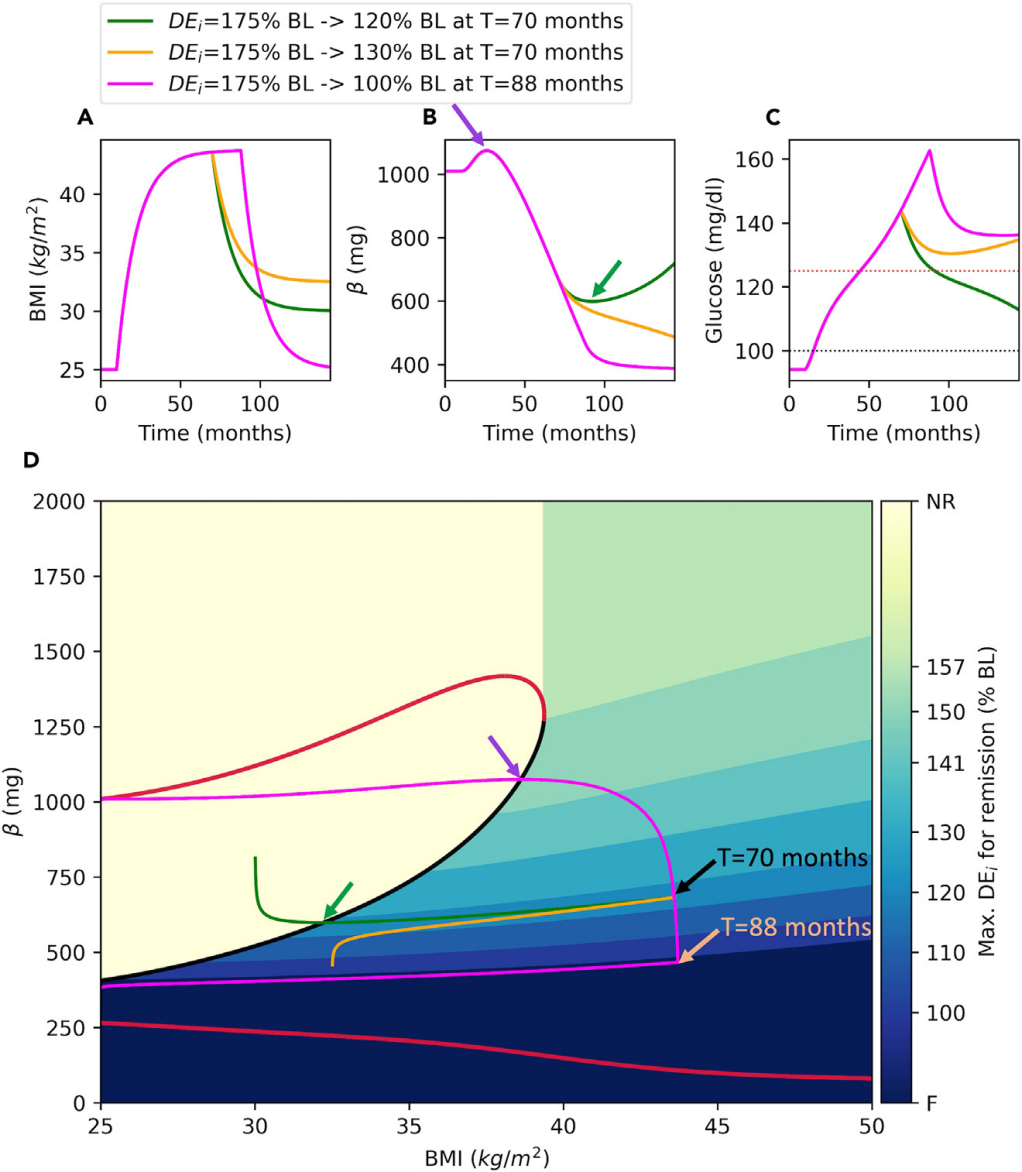
Bifurcation diagram with calorie restriction boundaries BMI (A), β (B) and Glucose (C) time courses are shown for different parameter sets. (D) Solution trajectories are superimposed onto the bifurcation diagram along with decision boundaries that show the necessary calorie restriction for remission. For green simulation, DE_i_ is increased by 175% over the baseline (BL) at T = 10 months and set back to 120% of the BL at T = 70 months. For orange simulation, DE_i_ is increased by 175% over the baseline (BL) at T = 10 months and set back to 130% of the BL at T = 70 months. For magenta simulation, DE_i_ is increased by 175% over the baseline (BL) at T = 10 months and set back to the BL at T = 88 months. Purple and green arrows in panels B and D show the peak and nadir of green trajectory from different perspectives, respectively. In panel C, black and red horizontal dotted lines mark the prediabetes (100 mg/dL) and diabetes (125 mg/dL) glucose cut offs. The color bar shows the maximum *DE*_*i*_ levels relative to baseline (percentage of BL) necessary for successful remission within the corresponding region. BL: baseline; NR: no restriction; F: failure.

Running outputs function with fn = 13 creates Figure 13.

> outputs(fn=13)

Figure 13 shows simulations for 3 different scenarios. For green simulation, DE_i_ is increased by 175% over the baseline (BL) at T = 10 months and set back to 120% of the BL at T = 70 months. For orange simulation, *DE*_*i*_ is increased by 175% over the baseline (BL) at T = 10 months and set back to 130% of the BL at T = 70 months. For magenta simulation, *DE*_*i*_ is increased by 175% over the baseline (BL) at T = 10 months and set back to the BL at T = 88 months. In green simulation, the remission is achieved because the *DE*_*i*_ is reduced to 120% of the baseline, while the phase point is inside the region marked by the 120% BL. On the other hand, at this same point, reducing *DE*_*i*_ to 130% of the baseline fails in remission, since this intervention cannot push the phase point to the left side of the threshold (Figure 13D, orange). Finally setting *DE*_*i*_ to 100% BL after 88 months fails in remission as well (Figure 13, magenta). Because at this point, the phase point has already entered the dark blue region marked by failure (Figure 13D, magenta).

***Note:*** In outputs function you may set fn = ’custom’ to create simulations with custom parameter values. In this case, you should also set the following objects for the desired parameter values;

it1: The time point to initiate the increase in daily energy intake over the baseline (days, the default is 300 days).

inc_i1s: The list of increase in daily energy intake over the baseline (the default is [0.7, 0.75]).

it2s: The list of intervention time points (the default is [2100,2400] days).

inc_i2s: The list daily energy intake at the intervention time-point (it2) calculated over the baseline (the default is [0.5, 0.2]). An inc_i2 value above inc_i1 indicates a further increase in daily energy intake at it2, and weight gain.

dec_b: the option whether to draw the color map (the default is False).

labels: The labels for each simulation (the default is a list of simulation number, where a list of strings should be provided for custom labels).

***Note:*** Running the main method in main.py script in KRT generates all the Python figures and animations.

## Limitations

This protocol describes how to use bifurcation analysis to understand the slow dynamics of the emergence and progression of diabetes with obesity using a recent model.^1^ However, the protocol and provided software can be used to analyze any models that use ordinary differential equations to describe the system’s dynamics. Although the software packages were designed and described with general use in mind, the software might need to be adjusted for different use cases. The other limitation of the protocol is that it gives best results if system variables operate on different timescales. This is particularly true for high dimensional models, since a two-dimensional projection of the bifurcation diagram is studied for the analysis. A larger separation between the time scales of the system variables makes analysis and results more robust.

## Troubleshooting

### Problem 1

**Figure 14:**
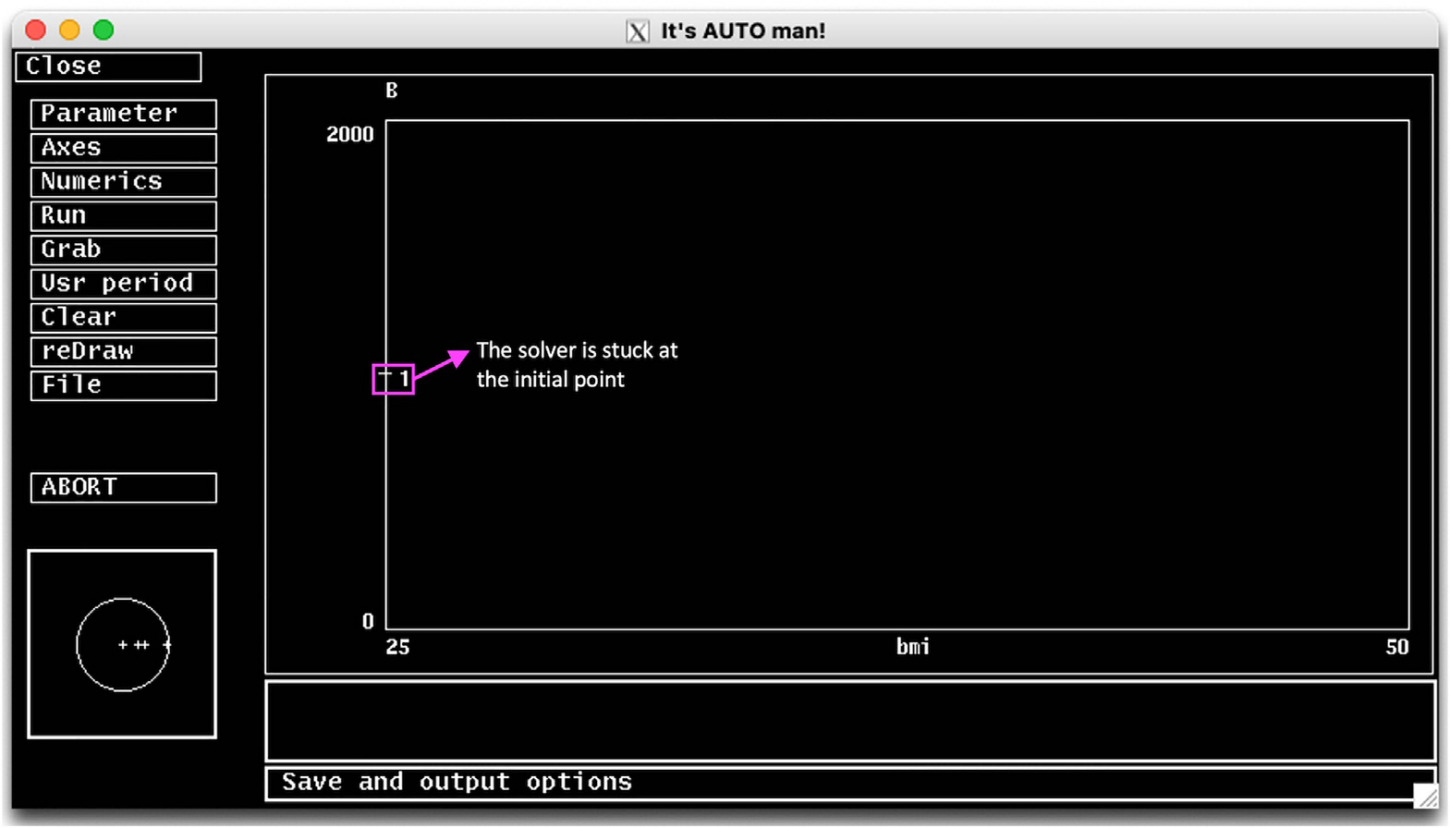
AUTO fails to converge if it is not started from a steady state

The AUTO needs to be initiated from a steady state to keep track of the steady state solutions as the bifurcation parameter changed in either direction. AUTO uses the initial conditions set in XPP as the starting point. Therefore, initial conditions in XPP must be set to a steady state. If AUTO is initiated from a point that is far from a steady state, the convergence fails and a single point appears in AUTO simulation screen (Figure 14) (related to Step 11).

### Potential solution

• Go to the XPP window.
• Switch the y-axis on the main simulation screen to B using Viewaxes menu, and set the axes limits.
• Press P on the keyboard to allow parameter input. Type in bmi and press enter. Now type in the desired value for bmi.

***Note:*** You may also click on Param on the Quick Access Menu on XPP, which lists all parameters and their values. Set bmi to desired value and click Go to initiate the simulation.

• Run the simulation by choosing Initialcond > (G)o (or press I > G on keyboard).
• Then repeat the simulation using the last point from previous simulation as new initial condition by choosing Initialcond > (L)ast (or press I > L on keyboard).
• Repeat the last step a few times until simulation results in a horizontal line, indicating variable does not change and system is at equilibrium.
• Go back to AUTO, and go to File > Reset Diagram to restart the process.

***Note:*** Restart Diagram option resets the AUTO but last image might remain on the simulation screen. You may erase the simulation screen by selecting Clear option on the main Menu on AUTO.

### Problem 2

**Figure 15:**
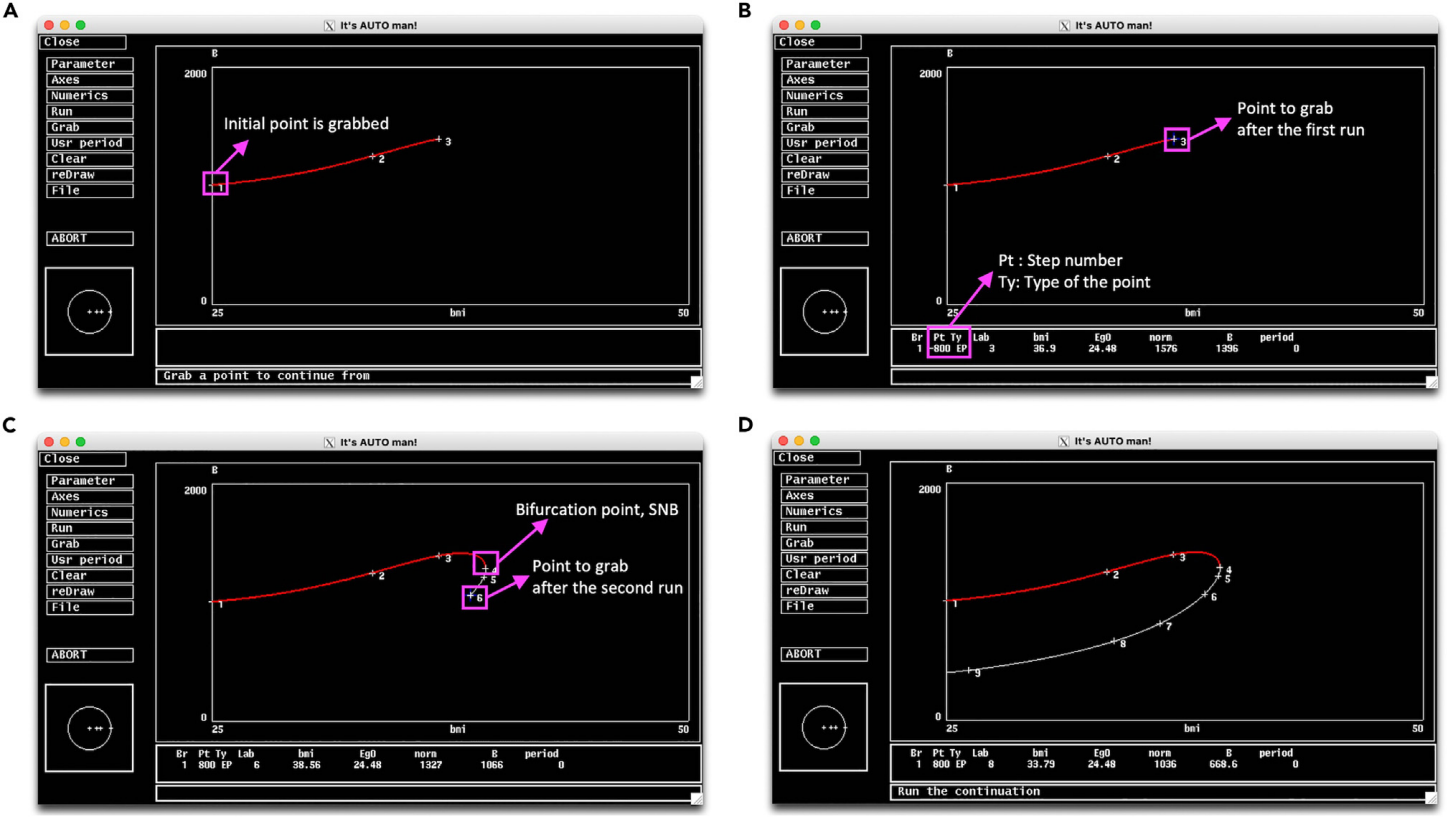
AUTO stops if it exceeds maximum step size Nmax (A) Pressing Grab a blue cross appears on the initial point. (B) Moving blue cross to the end of the curve (EP) to grab that point. (C) Restarting AUTO from the end of the first run AUTO continues for another Nmax. Curve takes a turn at a saddle node bifurcation (SNB). (D) Restarting AUTO from the end of the second run with a negative Ds to have bifurcation parameter to change in negative direction. AUTO completes the branch after 3 grabs and runs.

If AUTO exceeds the maximum number of steps (Nmax) and stops prematurely before completing a solution branch (Figure 15A) you may continue from where it left by using the Grab option (related to Step 11). To better describe this problem and possible solution, we set Nmax to 800, a relatively small value for following illustration.

### Potential solution

• Click the Grab button on the menu, and a blue cross will appear on the initial point (Figure 15A). This allows you to navigate through the branch. You can use the arrow keys to move on step at a time or you can press tab to jump through Npr number of steps set in Numerics.
• Move the blue cross to the end of the branch. Notice that information down the screen will change as you move the blue cross through the curve. When you reach to the end of the curve, Ty will say EP (Figure 15B), which stands for end point indicating the last point of a run.
• Press enter to grab the EP and then click Run for AUTO to continue.
• AUTO takes another Nmax steps and stops again (Figure 15C). Notice system goes through a saddle node bifurcation (SNB), where curve turns down and leftward.
• Click Grab and move blue cross to the end of the curve. On the Numerics menu set Ds to a negative value to tell AUTO to move bifurcation point at the negative direction. You may also increase Nmax to let AUTO to finish at one final go (Figure 15D).

### Problem 3

When generating a second branch of steady states AUTO may fail in converging to a steady state. This is usually caused by previously grabbed point kept in memory (related to Step 12e).

### Potential solution

If the **grab** function in AUTO has been used in earlier steps, the **grab** must be cleared before you continue with generating other branches. In that case, you must reset the grabbed point first before you continue. For this, go to the File menu on AUTO and choose **Clear Grab** option.

### Problem 4

The create_script function reads the user defined functions in XPP and translates them into Python *lambda* functions (related to step 15f). In the XPPAUT ode file, any object can be used in a statement before being declared. However, in Python, an object can only be used after being declared. The order of the user defined functions in XPP or the way they are nested may cause errors in Python. In that case, Python raises an ‘*undefined function or variable’* error referring to the user defined functions in XPP.

### Potential solution

Open the *model.py* file and locate the function call that is causing the error. Manually reorder the variables and make sure the variables used in the function are declared properly.

### Problem 5

If the protocol is run on an interactive python notebook such as Jupyter Notebook, the animations may not run or some figures may not appear appropriately inline (related to step 18).

### Potential solution

Before running the functions that generates the figures or animations, run following magic command on Jupyter Notebook to tell Python to create figures on separate windows.

> %matplotlib qt

## Resource availability

### Lead contact

Further information and requests for resources and data should be directed to and will be fulfilled by the lead contact, Vehpi Yildirim (v.yildirim@amsterdamumc.nl).

### Technical contact

Further information requests about technical details should be directed to and will be fulfilled by the technical contact, Vehpi Yildirim (v.yildirim@amsterdamumc.nl).

### Materials availability

This study did not generate any reagents.

### Data and code availability

All datasets and codes generated during this study are available online under MIT license at GitHub: https://zenodo.org/doi/10.5281/zenodo.10525256.
